# Supplementary material for: Incorporating reef fish avoidance behavior improves accuracy of species distribution models
Source: PeerJ. 2020 Jun 3;8:e9246. doi: 10.7717/peerj.9246 (PMC7275679; doi:10.7717/peerj.9246)
Supplement: Table S1 [file peerj-08-9246-s001.docx]

Supplemental Table S1:

**List of reef fish species targeted by spearfishing in Hawai**‘**i and recorded on diver operated stereo video (stereo-DOV) surveys.**

| **Family** | **Species** |
| --- | --- |
| Acanthuridae | *Acanthurus achilles* |
|  | *Acanthurus blochii* |
|  | *Acanthurus dussumieri* |
|  | *Acanthurus guttatus* |
|  | *Acanthurus leucopareius* |
|  | *Acanthurus nigroris* |
|  | *Acanthurus olivaceus* |
|  | *Acanthurus triostegus* |
|  | *Acanthurus xanthopterus* |
|  | *Ctenochaetus strigosus* |
|  | *Naso lituratus* |
|  | *Naso unicornis* |
|  | *Zebrasoma veliferum* |
| Carangidae | *Carangoides ferdau* |
|  | *Carangoides orthogrammus* |
|  | *Caranx melampygus* |
| Kyphosidae | *Kyphosus sp* |
| Labridae | *Anampses cuvier* |
|  | *Bodianus bilunulatus* |
| Lethrinidae | *Monotaxis grandoculis* |
| Lutjanidae | *Lutjanus fulvus* |
|  | *Lutjanus kasmira* |
| Mullidae | *Mulloidichthys flavolineatus* |
|  | *Mulloidichthys vanicolensis* |
|  | *Parupeneus cyclostomus* |
|  | *Parupeneus insularis* |
|  | *Parupeneus multifasciatus* |
|  | *Parupeneus pleurostigma* |
| Scaridae | *Calotomus carolinus* |
|  | *Chlorurus perspicillatus* |
|  | *Chlorurus sordidus* |
|  | *Scarus psittacus* |
|  | *Scarus rubroviolaceus* |
| Serranidae | *Cephalopholis argus* |
