# Supplementary material for: Incorporating reef fish avoidance behavior improves accuracy of species distribution models
Source: PeerJ. 2020 Jun 3;8:e9246. doi: 10.7717/peerj.9246 (PMC7275679; doi:10.7717/peerj.9246)
Supplement: Table S2 [file peerj-08-9246-s002.docx]

Supplemental Table S2:

**Description of habitat predictors used in species distribution models (SDMs).**

| **Type** | **Dataset** | **Description** | **Units** |
| --- | --- | --- | --- |
| **Seafloor Topography** | Depth 60m | Mean seafloor depth | m |
|  | Slope 60, 240m | Mean maximum rate of change in seafloor depth between each grid cell and its neighbors | Degree |
|  | Slope of Slope 60, 240m | Mean maximum rate of change in seafloor slope between each grid cell and its neighbors | Degree |
|  | Aspect 60m | Downslope direction of maximum rate of change in seafloor depth between each grid cell and its neighbors: Sine circular mean, cosine circular mean, circular SD | Unitless |
|  | Planar curvature 60m | Mean seafloor curvature perpendicular to the line of maximum slope; value indicates whether flow will converge or diverge over a point; values can be - (concave), + (convex), or 0 (flat) | Radians/m |
|  | Profile curvature 60m | Mean seafloor curvature along the line of maximum slope; value indicates whether flow will accelerate or decelerate over the curve; values can be + (concave), - (convex), or 0 (flat) | Radians/m |
|  | Bathymetric position index (BPI) 60, 240m | Mean difference in seafloor depth and the mean seafloor depth in an annular neighborhood of specified inner and outer radii; values indicate a location's position relative to the surrounding area; values can be + (ridges), - (valleys), or 0 (flat areas or areas of constant slope) | m |
| **Benthic Habitat composition** | Percentage of landscape 60m | Percent of grid cell (60m) composed of each cover type: Coral, CCA, Macroalgae, Turf, Soft bottom | Percent |
|  | Proximity  index 60m | Values indicate the spatial context of both the degree of patch isolation and degree of seascape fragmentation; a value of 0 indicates no neighbors of the same cover class within the search radius, and values increase as patches of the same class become more numerous, closer, and more contiguous | Unitless |
|  | Shannon's diversity index 60m | Values indicate habitat diversity in terms of the number of different cover classes present (richness) and the proportional distribution of area among the different cover classes (evenness); a value of 0 indicates only 1 patch (no diversity) and values increase as either the number of different cover classes increases or area becomes more evenly distributed among classes | Unitless |
| **Geographic** | Latitude | Latitude at each model grid cell centroid | Meters |
|  | Longitude | Longitude at each model grid cell centroid | Meters |
|  | Distance to shore | Straight line (Euclidean) distance to the shoreline | Meters |
| **Wave energy** | Wave power 60m | Mean wave power (wave height x wave period) derived from a 1 year (2000-2009) hindcast wave model | Kilowatts per meter |

*For predictor generation methods see Stamoulis, K. A., M. Poti, J. M. S. Delevaux, M. K. Donovan, A. M. Friedlander, and M. S. Kendall. 2016. Chapter 4: Fishes - Reef Fish. Pages 156–196 *in* B. M. Costa and M. S. Kendall, editors. Marine Biogeographic Assessment of the Main Hawaiian Islands. Bureau of Ocean Energy Management and National Oceanic and Atmospheric Administration, Silver Spring, M.D.
